# Supplementary material for: A fault-intrusion-tolerant system and deadline-aware algorithm for scheduling scientific workflow in the cloud
Source: PeerJ Comput Sci. 2021 Nov 2;7:e747. doi: 10.7717/peerj-cs.747 (PMC8576554; doi:10.7717/peerj-cs.747)
Supplement: Supplemental Information 6 [file peerj-cs-07-747-s006.zip › Myproject1.1/docs/index-files/index-13.html]

N-Index


JavaScript is disabled on your browser.


- Overview
- Package
- Class
- Use
- Tree
- Deprecated
- Index
- Help

- Prev Letter
- Next Letter

- Frames
- No Frames

- All Classes

A B C D E F G H I J L M N O P Q R S T U V W Z 


## N

NetDatacenterBroker - Class in org.cloudbus.cloudsim.network.datacenter
:   NetDatacentreBroker represents a broker acting on behalf of Datacenter provider.

NetDatacenterBroker(String) - Constructor for class org.cloudbus.cloudsim.network.datacenter.NetDatacenterBroker
:   Created a new DatacenterBroker object.

Network\_Event\_DOWN - Static variable in class org.cloudbus.cloudsim.core.CloudSimTags


Network\_Event\_Host - Static variable in class org.cloudbus.cloudsim.core.CloudSimTags


Network\_Event\_send - Static variable in class org.cloudbus.cloudsim.core.CloudSimTags


Network\_Event\_UP - Static variable in class org.cloudbus.cloudsim.core.CloudSimTags


NetworkCloudlet - Class in org.cloudbus.cloudsim.network.datacenter
:   NetworkCloudlet class extends Cloudlet to support simulation of complex applications.

NetworkCloudlet(int, long, int, long, long, long, UtilizationModel, UtilizationModel, UtilizationModel) - Constructor for class org.cloudbus.cloudsim.network.datacenter.NetworkCloudlet


NetworkCloudletSpaceSharedScheduler - Class in org.cloudbus.cloudsim.network.datacenter
:   CloudletSchedulerSpaceShared implements a policy of scheduling performed by a virtual machine.

NetworkCloudletSpaceSharedScheduler() - Constructor for class org.cloudbus.cloudsim.network.datacenter.NetworkCloudletSpaceSharedScheduler
:   Creates a new CloudletSchedulerSpaceShared object.

NetworkConstants - Class in org.cloudbus.cloudsim.network.datacenter


NetworkConstants() - Constructor for class org.cloudbus.cloudsim.network.datacenter.NetworkConstants


NetworkDatacenter - Class in org.cloudbus.cloudsim.network.datacenter
:   NetworkDatacenter class is a Datacenter whose hostList are virtualized and networked.

NetworkDatacenter(String, DatacenterCharacteristics, VmAllocationPolicy, List<Storage>, double) - Constructor for class org.cloudbus.cloudsim.network.datacenter.NetworkDatacenter
:   Allocates a new NetworkDatacenter object.

NetworkExample1 - Class in org.cloudbus.cloudsim.examples.network
:   A simple example showing how to create
    a datacenter with one host and a network
    topology and and run one cloudlet on it.

NetworkExample1() - Constructor for class org.cloudbus.cloudsim.examples.network.NetworkExample1


NetworkExample2 - Class in org.cloudbus.cloudsim.examples.network
:   A simple example showing how to create
    two datacenters with one host and a
    network topology each and run two cloudlets
    on them.

NetworkExample2() - Constructor for class org.cloudbus.cloudsim.examples.network.NetworkExample2


NetworkExample3 - Class in org.cloudbus.cloudsim.examples.network
:   A simple example showing how to create
    two datacenters with one host each and
    run cloudlets of two users with network
    topology on them.

NetworkExample3() - Constructor for class org.cloudbus.cloudsim.examples.network.NetworkExample3


NetworkExample4 - Class in org.cloudbus.cloudsim.examples.network
:   A simple example showing how to create
    a datacenter with one host and a network
    topology and and run one cloudlet on it.

NetworkExample4() - Constructor for class org.cloudbus.cloudsim.examples.network.NetworkExample4


NetworkHost - Class in org.cloudbus.cloudsim.network.datacenter
:   NetworkHost class extends Host to support simulation of networked datacenters.

NetworkHost(int, RamProvisioner, BwProvisioner, long, List<? extends Pe>, VmScheduler) - Constructor for class org.cloudbus.cloudsim.network.datacenter.NetworkHost


NetworkPacket - Class in org.cloudbus.cloudsim.network.datacenter
:   NewtorkPacket represents the packet which travel from one server to another.

NetworkPacket(int, HostPacket, int, int) - Constructor for class org.cloudbus.cloudsim.network.datacenter.NetworkPacket


NetworkTopology - Class in org.cloudbus.cloudsim
:   NetworkTopology is a class that implements network layer in CloudSim.

NetworkTopology() - Constructor for class org.cloudbus.cloudsim.NetworkTopology


NetworkVm - Class in org.cloudbus.cloudsim.network.datacenter
:   NetworkVm class extends Vm to support simulation of networked datacenters.

NetworkVm(int, int, double, int, int, long, long, String, CloudletScheduler) - Constructor for class org.cloudbus.cloudsim.network.datacenter.NetworkVm


NetworkVmAllocationPolicy - Class in org.cloudbus.cloudsim.network.datacenter
:   NetworkVmAllocationPolicy is an VmAllocationPolicy that chooses, as the host for a VM, the host
    with less PEs in use.

NetworkVmAllocationPolicy(List<? extends Host>) - Constructor for class org.cloudbus.cloudsim.network.datacenter.NetworkVmAllocationPolicy
:   Creates the new VmAllocationPolicySimple object.

NextCycle - Static variable in class org.cloudbus.cloudsim.core.CloudSimTags


nextIdx - Static variable in class org.cloudbus.cloudsim.NetworkTopology


nexttime - Static variable in class org.cloudbus.cloudsim.network.datacenter.NetworkConstants


NonPowerAware - Class in org.cloudbus.cloudsim.examples.power.planetlab
:   A simulation of a heterogeneous non-power aware data center: all hosts consume maximum power all
    the time.

NonPowerAware() - Constructor for class org.cloudbus.cloudsim.examples.power.planetlab.NonPowerAware


NonPowerAware - Class in org.cloudbus.cloudsim.examples.power.random
:   A simulation of a heterogeneous non-power aware data center: all hosts consume maximum power all
    the time.

NonPowerAware() - Constructor for class org.cloudbus.cloudsim.examples.power.random.NonPowerAware


NOT\_REGISTERED - Static variable in class org.cloudbus.cloudsim.File
:   Denotes that this file has not been registered to a Replica Catalogue

NUMBER\_OF\_HOSTS - Static variable in class org.cloudbus.cloudsim.examples.power.planetlab.PlanetLabConstants


NUMBER\_OF\_HOSTS - Static variable in class org.cloudbus.cloudsim.examples.power.random.RandomConstants


NUMBER\_OF\_VMS - Static variable in class org.cloudbus.cloudsim.examples.power.random.RandomConstants


numbervm - Variable in class org.cloudbus.cloudsim.network.datacenter.AppCloudlet


numEventsWaiting(Predicate) - Method in class org.cloudbus.cloudsim.core.SimEntity
:   Count how many events matching a predicate are waiting in the entity's deferred queue.

numEventsWaiting() - Method in class org.cloudbus.cloudsim.core.SimEntity
:   Count how many events are waiting in the entity's deferred queue.

numport - Variable in class org.cloudbus.cloudsim.network.datacenter.Switch


numStage - Variable in class org.cloudbus.cloudsim.network.datacenter.NetworkCloudlet

A B C D E F G H I J L M N O P Q R S T U V W Z

- Overview
- Package
- Class
- Use
- Tree
- Deprecated
- Index
- Help

- Prev Letter
- Next Letter

- Frames
- No Frames

- All Classes
